# Iron Acquisition Proteins of *Pseudomonas aeruginosa* as Potential Vaccine Targets: In Silico Analysis and In Vivo Evaluation of Protective Efficacy of the Hemophore HasAp

**DOI:** 10.3390/vaccines11010028

**Published:** 2022-12-23

**Authors:** Abdelrahman S. Hamad, Eva A. Edward, Eman Sheta, Hamida M. Aboushleib, Mohammed Bahey-El-Din

**Affiliations:** 1 Department of Microbiology and Immunology, Faculty of Pharmacy, Alexandria University, Alexandria P.O. Box 25435, Egypt; 2 Pathology Department, Faculty of Medicine, Alexandria University, Alexandria P.O. Box 21131, Egypt

**Keywords:** *Pseudomonas aeruginosa*, vaccine, iron acquisition proteins, HasAp, naloxone

## Abstract

Background: *Pseudomonas aeruginosa* (PA) is a Gram-negative pathogen responsible for fatal nosocomial infections worldwide. Iron is essential for Gram-negative bacteria to establish an infection. Therefore, iron acquisition proteins (IAPs) of bacteria are attractive vaccine targets. Methodology: A “Reverse Vaccinology” approach was employed in the current study. Expression levels of 37 IAPs in various types of PA infections were analyzed in seven previously published studies. The IAP vaccine candidate was selected based on multiple criteria, including a high level of expression, high antigenicity, solubility, and conservation among PA strains, utilizing suitable bioinformatics analysis tools. The selected IAP candidate was recombinantly expressed in *Escherichia coli* and purified using metal affinity chromatography. It was further evaluated in vivo for protection efficacy. The novel immune adjuvant, naloxone (NAL), was used. Results and discussion: HasAp antigen met all the in silico selection criteria, being highly antigenic, soluble, and conserved. In addition, it was the most highly expressed IAP in terms of average fold change compared to control. Although HasAp did excel in the in silico evaluation, subcutaneous immunization with recombinant HasAp alone or recombinant HasAp plus NAL (HasAP-NAL) did not provide the expected protection compared to controls. Immunized mice showed a low IgG2a/IgG1 ratio, indicating a T-helper type 2 (Th2)-oriented immune response that is suboptimal for protection against PA infections. Surprisingly, the bacterial count in livers of both NAL- and HasAp-NAL-immunized mice was significantly lower than the count in the HasAp and saline groups. The same trend was observed in kidneys and lungs obtained from these groups, although the difference was not significant. Such protection could be attributed to the enhancement of innate immunity by NAL. Conclusions: We provided a detailed in silico analysis of IAPs of PA followed by in vivo evaluation of the best IAP, HasAp. Despite the promising in silico results, HasAp did not provide the anticipated vaccine efficacy. HasAp should be further evaluated as a vaccine candidate through varying the immunization regimens, models of infection, and immunoadjuvants. Combination with other IAPs might also improve vaccination efficacy. We also shed light on several highly expressed promising IAPs whose efficacy as vaccine candidates is worthy of further investigation.

## 1. Introduction

*Pseudomonas aeruginosa* (PA) is a Gram-negative, nosocomial bacterial pathogen responsible for severe infections in patients who are immunosuppressed, intubated, or ventilated. It is primarily associated with burn victims and cystic fibrosis (CF) patients [1]. Infections due to this pathogen are difficult to treat because of both intrinsic and acquired resistance to multiple groups of antimicrobial agents [2]. In fact, several studies have reported pan-drug-resistant PA among hospitalized patients [3,4]. A horrible scenario would be that these pan-drug-resistant strains dominate, thus sending the world back to the pre-antibiotic era [5]. Therefore, research focus on vaccination is an essential strategy which needs to be performed in parallel with new antibiotic discovery to win the battle against this pathogen.

PA is armed with a multitude of virulence factors including proteases, lipases, exotoxin A, and a type III secretion system [6]. Iron acquisition from the host has been proven to be essential for Gram-negative pathogens to establish an infection, and PA is a prime example [7]. Iron is an essential cofactor for respiratory and DNA synthesizing enzymes [8]. Moreover, it aids in the construction of mature biofilms in airways of CF patients, which are responsible for conferring resistance to various antibiotics and immune defenses [9]. In fact, iron scarcity forces PA to initiate twitching motility, which disrupts its biofilm structure [10]. 

PA is equipped with an armory of iron pirating systems, including siderophores (ferric binders) [11], heme acquisition proteins (hemophores) [12], and the Feo system specialized in ferrous uptake [13]. Both siderophores and hemophores are internalized via surface receptors embedded in the outer membrane called “TonB-dependent transporters or TBDTs”. These receptors depend on the ExbB-ExbD-TonB system, which transfers the energy generated from the proton motive force of the cytoplasmic membrane through the periplasm to these receptors [11]. TBDTs mainly transport iron complexes, but they can also transport other metals such as zinc and nickel [14].

During the course of infection, PA shows a smart adaptability in choosing the best iron acquisition tool [15]. Pyoverdines are the major high-affinity siderophores that bind Fe^3+^ mainly during the acute phase of infection [16]. They are able to displace iron from transferrin and participate in biofilm formation [17]. Pyoverdines also function as signaling molecules triggering the release of the tissue-damaging PrpL protease and exotoxin A [18]. Three heme uptake systems, Has, Phu, and Hxu, are employed to obtain heme from circulating hemoglobin and hemopexin. The Has system employs a messenger protein, HasAp, that grabs heme back to a receptor termed, HasR. Phu and Hxu systems, on the other hand, interact directly with heme via their surface receptors, PhuR and HxuA, respectively [19]. In addition, PA can utilize multiple siderophores secreted by other microbes, “xenosiderophores” [15,20]. 

The traditional approach of sub-unit vaccine design for a certain pathogen involves several steps. Initially, the pathogen is cultivated and then certain proteins are purified without prior knowledge about their immunogenicity. Finally, assessment of the protective efficacy is conducted through in vivo animal trials. However, this approach is only applicable if the pathogen in question is cultivable, and the antigen is expressed in large quantities suitable for vaccine development. In addition, it could detect antigens expressed only during in vitro cultivation, but not the ones expressed in vivo during infection. However, thanks to the advent of whole genome sequencing technologies, the whole proteome of a pathogen can now be screened for promising protective antigens using in silico immunoinformatics tools followed by expression of the candidates and confirming their immunogenicity through in vivo experiments, a process termed “reverse vaccinology” [21]. The latter approach is free from the pitfalls pertinent to the conventional approach. Moreover, it saves time, money, and effort that would otherwise be wasted in unworthy experimental trials [22,23].

The efficacy of several PA iron acquisition proteins—hereafter termed (IAPs)—as vaccine targets has been previously tested [24,25,26,27]. However, as far as we know, this is the first study to employ a systematic, inverted pyramid vaccine target selection approach, focusing only on IAPs and involving several selection criteria. The literature was searched for all IAPs, followed by classification of IAPs based on iron acquisition mechanism. Then, seven published transcriptomic analyses were screened to choose the most highly expressed IAPs [25,28,29,30,31,32,33]. Furthermore, IAPs were subjected to extensive in silico analysis, whereby antigenicity, water solubility, conservation among PA strains, and homology to human and mouse proteins were assessed. Finally, the antigen with the best scoring was recombinantly expressed and subjected to in vivo assessment in combination with the novel immunoadjuvant, naloxone (NAL) [34].

## 2. Materials and Methods

### 2.1. Bioinformatic In Silico Selection of the Best Iron Acquisition Vaccine Candidate

All iron-regulated proteins of PA PAO1 strain were searched for and classified using the GenBank database at https://www.ncbi.nlm.nih.gov/nuccore/NC_002516# (accessed on 1 February 2021), as well as previously published research [20,30,32]. Cytoplasmic and inner membrane proteins were excluded using the subcellular localization feature in https://www.pseudomonas.com# (accessed on 3 February 2021) [35], thereby ensuring surface exposure and accessibility to the immune system. Published transcriptomic analyses were screened to assess the level of expression of iron acquisition proteins of *P. aeruginosa* [25,28,29,30,31,32,33]. The average fold change across these studies was used as a metric to rank the selected proteins. SignalP 4.1 [36] at http://www.cbs.dtu.dk/services/SignalP-4.1/ (accessed on 5 February 2021) was used to predict signal peptides within protein sequences which were subsequently removed. I-Tasser tool [37] at http://zhanglab.ccmb.med.umich.edu/I-TASSER/ (accessed on 5 February 2021) was used to predict 3D structures from raw amino acid sequences. The model with the highest confidence score (C-score) was selected [38]. Whole-protein antigenicity was predicted from raw protein sequences using Antigenic [39] at http://77.68.43.135:8080/Antigenic (accessed on 6 February 2021) and AntigenPro [40] at http://scratch.proteomics.ics.uci.edu/ (accessed on 6 February 2021). The average of the scores from both tools was calculated, and a decision threshold of 0.5 was considered. SOlart, a structure-based solubility prediction tool available at http://babylone.ulb.ac.be/SOLART (accessed on 7 February 2021) [41], was used. Proteins whose solubility scores exceeded 0.5 were subsequently chosen. The BLAST-P program, available at https://blast.ncbi.nlm.nih.gov/Blast.cgi?PAGE=Proteins (accessed on 12 February 2021), was used to BLAST antigen sequences against non-redundant protein database of *P. aeruginosa* [42,43]. The “maximum number of aligned sequences to display” option was adjusted to 500. Models as well as uncultured and environmental sequences were excluded. For each antigen, alignment data were downloaded using the NCBI multiple sequence alignment viewer tool. Then, the mean of (percentage identity X query coverage), hereby designated as IC, was used as a metric to compare the conservation of antigens [23]. A threshold value of 90% was considered. The BLAST-P program was also used to check the similarity of the selected *P. aeruginosa* antigens to proteins from human and mouse proteomes [23,42,43]. Hits of each antigen were sorted using the same previously mentioned sorting metrics. Four lists were created which included proteins most highly expressed, most antigenic, most soluble, and most conserved/least similar to human or mouse proteins. Venn diagram data analysis was used to determine the intersection between these lists, i.e., the candidate antigen(s). 

### 2.2. Cloning and Expression of HasAp

For polymerase chain reaction (PCR), the forward primer (TATAGGATCC**G**ATGAGCATTTCGATTTCCTACAGC) and reverse primer (TATAAAGCTTTCACGCCAGGGCCAGGTCG) were designed based on the published *hasAp* gene sequence from the PAO1 strain (NCBI nucleotide reference sequence number: NC_002516.2). Recognition sites (underlined) of BamHI and HindIII were added to the forward and reverse primers, respectively, to facilitate cloning. An extra guanine was inserted in the forward primer (in bold) to adjust the reading frame for cloning in pQE31 plasmid (Qiagen, Hilden, Germany) to add an N-terminal His tag. PCR amplification of *hasAp* gene was carried out using Phusion™ High-Fidelity DNA Polymerase (Thermo Scientific™, Waltham, MA, USA) where genomic DNA of *P. aeruginosa* PAO1 was employed as a template. The *hasAp* amplicon and pQE31 plasmid were digested with BamHI and HindIII restriction enzymes (New England Biolabs, Ipswich, MA, USA) following the manufacturer’s instructions. This was followed by ligation using T4 DNA Ligase (New England Biolabs, Ipswich, MA, USA). *E. coli* M15 (pREP4) was subsequently transformed with the pQE31-*hasAp* construct.

Expression of *hasAp* was carried out as previously described [44] with slight modifications. Briefly, an overnight culture of *E. coli* M15 (pREP4) (pQE31-*hasAp*) was made in Luria Bertani (LB) medium containing ampicillin and kanamycin (200 μg/mL and 25 μg/mL, respectively) at 37 °C with vigorous shaking. Fresh 200 mL LB medium supplemented with the same antibiotics was inoculated with 4 mL from the overnight culture and incubation was continued at 37 °C till an OD of 0.8. Centrifugation was performed at 6000× *g* for 10 min at 4 °C. Supernatant was discarded and pellet was resuspended in an equal volume of M9 minimal medium formulated as previously described [45] but without vitamins, micronutrients, or glucose and was supplemented with the same antibiotics. Incubation was continued for 30 min at 37 °C then the temperature was lowered to 30 °C and isopropyl β-D-thiogalactopyranoside (IPTG) (Melford, U.K.) was added at a final concentration of 1 mM for 22 h. Cells were centrifuged using the same previously mentioned settings and the pellet was stored at −20 °C until needed.

Cell lysis and purification using Ni-NTA metal affinity chromatography under native conditions were performed as described in protocols 9 and 12 of the QIAexpressionist handbook [46]. Purified HasAp was analyzed by sodium dodecyl sulfate–polyacrylamide gel electrophoresis (SDS-PAGE). Imidazole was removed via a desalting PD-10 column (GE Healthcare, Buckinghamshire, U.K.) using phosphate-buffered saline (PBS) as the eluant. Endotoxins were removed using polymyxin B sulfate agarose beads (Sigma-Aldrich, Prod. No. P1411, St. Louis, MO, USA) as per the manufacturer’s instructions, where the buffer used for washing, equilibration, and elution was pyrogen-free PBS pH 7.8. The Pyrotell^®^ Limulus Amebocyte Lysate (LAL) test (Associates of CAPE COD Inc., East Falmouth, MA, USA) confirmed the absence of endotoxins.

### 2.3. Western Blot Analysis

Western blot analysis was performed as previously described [47]. Briefly, HasAp was purified using SDS-PAGE followed by transfer from gel to a nitrocellulose membrane which was then incubated in a blocking solution (5% skimmed milk in Tris-buffered saline (TBS)) overnight at 4 °C. Blocking solution was discarded, followed by washing twice with TBST (Tris-buffered saline/0.05% Tween 20) for 10 min each while shaking at room temperature (RT). Next, the membrane was incubated with primary antibody at RT for 1 h. The primary antibody consisted of either mouse anti-His tag antibody (Biolegend Co., San Diego, CA, USA) (diluted 1:2500 in blocking solution) or sera from mice immunized intraperitoneally (i.p.) with sub-lethal doses (10^5^ CFU/mouse) of PA ATCC 9027 (diluted 1:100 in blocking solution). The primary antibody was discarded, and the membrane was washed twice with TBST for 10 min each at RT. The membrane was incubated with secondary antibody (horseradish peroxidase-labeled secondary antibody to mouse IgG) (KPL, Lexington, MA, USA) diluted 1:500 in blocking solution for 1 h at RT. Secondary antibody was discarded and the membrane was washed four times with TBST for 10 min each while shaking at RT. DAB (3,3′-diaminobenzidine tetrahydrochloride) (BioBasic Inc., Markham, ON, Canada) was dissolved in PBS (pH 7.5) to a final concentration of 0.5 mg/mL, then hydrogen peroxide was added (1 μL of 30% H_2_O_2_ per ml of DAB solution). The DAB solution was subsequently added to the membrane and incubated for 10–30 min until protein bands were visible.

### 2.4. Mice, Immunization Regimens, and Strains

Six-to-eight-week-old female Swiss albino mice (20–25 g) were purchased from Theodor Bilharz Research Institute, Giza, Egypt. All animal procedures were carried out according to the international and institutional guidelines and have been ethically approved by the Animal Care and Use Committee (ACUC) of the Faculty of Pharmacy, Alexandria University, Egypt (approval reference no. 0620223151118). The mice were divided into four groups (five mice/group) and adequate food and water were provided ad libitum. Mice of each group were injected subcutaneously (S.C.) with either HasAp plus naloxone (HasAp-NAL), HasAp alone, NAL alone, or saline according to the regimen shown in Table 1. The doses used were 10 μg/mouse for HasAp and 150 μg/mouse for NAL as previously described [48]. Serum samples were obtained, following blood collection using the submandibular vein method [49], before the first vaccine dose and two weeks after the last booster and were stored at −20 °C until needed.

### 2.5. Enzyme-Linked Immunosorbent Assay (ELISA)

Antigen-specific ELISA (ASE) was performed as previously described [50] with some modifications. Briefly, 96-well high-binding ELISA plates (Greiner Bio One, Frickenhausen, Germany) were coated (100 μL/well) overnight at 4 °C with 1 μg of either purified HasAp or HitA (irrelevant His-tagged protein as a control). Plates were washed three times with PBS and blocked with 5% skimmed milk in PBS for 3 h followed by washing three times using PBS. Serum samples were diluted 1:1000 in blocking buffer and incubated for 1 h at RT followed by washing 3 times with PBS. Horseradish peroxidase-labeled goat anti-mouse total IgG, IgG1, or IgG2a antibodies (KPL, Massachusetts, USA) were diluted in a ratio of 1:500 with blocking solution and 50 μL were added to each well followed by 1 h incubation at RT. The plate was washed six times with PBS; then, 100 μL of the TMB (3,3′,5,5′-tetramethylbenzidine) Microwell Peroxidase Substrate System (KPL, Massachusetts, USA) was added to each well. Color development was allowed by incubation in the dark for 10–15 min. Fifty microliters of 1M sulfuric acid were added to each well to stop the reaction, and absorbance was measured using a microplate reader (Biotek ELx800, Winooski, VT, USA) at 450 nm.

### 2.6. Bacterial Challenge and Bacterial Count in Homogenized Organs

Bacterial challenge and count were performed as previously described [50] with modifications. Two weeks after the last immunization, mice were challenged i.p. with a high sub-lethal dose (10^8^ CFU/mouse) of PA ATCC 9027. Mice were euthanized 48 h post-challenge where livers, lungs, and kidneys were collected, homogenized in sterile saline, serially diluted, and plated on cetrimide agar plates (Oxoid, Hampshire, U.K.). Plates were incubated at 37 °C for 24 h to determine the viable bacterial count in organs.

### 2.7. Histopathology

Parts of the livers were harvested from each of the sacrificed mice from each of the four groups and fixed for 24 h in formalin. They were processed as formalin-fixed paraffin blocks. Four-micron-thick sections were cut and stained by hematoxylin and eosin (H&E) stain. Slides were assessed and scored blindly for inflammatory changes. Portal inflammation and vascular congestion were scored from 0 to 3: 0 = absent, 1 = mild, 2 = moderate, and 3 = severe. Necroinflammatory foci or microabscesses were counted under a 100× power field. Then, they were scored according to the HAI-modified staging system: 0 = absent, 1 = one focus, 2 = 2–4 foci, 3 = 5–10 foci, or 4 if >10 foci were seen/100× power field [24,51].

### 2.8. Data Analysis and Statistics

Microsoft Excel (Office 365) was used to create the expression level heatmap showing the average fold change for each IAP. A Venn diagram was created using InteractiVenn tool available at http://www.interactivenn.net/index.html (accessed on 2 March 2021) [52]. Statistical analysis was performed using GraphPad Prism version 5.00 for Windows, GraphPad Software, San Diego, CA, USA, www.graphpad.com. Comparison between multiple groups was performed using one-way ANOVA with Tukey’s test for post hoc analysis. Kruskal–Wallis and Dunn’s multiple comparison tests were used in the analysis of histopathology data.

## 3. Results

### 3.1. In Silico Analysis

#### 3.1.1. Expression Level of Iron Acquisition Genes

**Figure 1:**
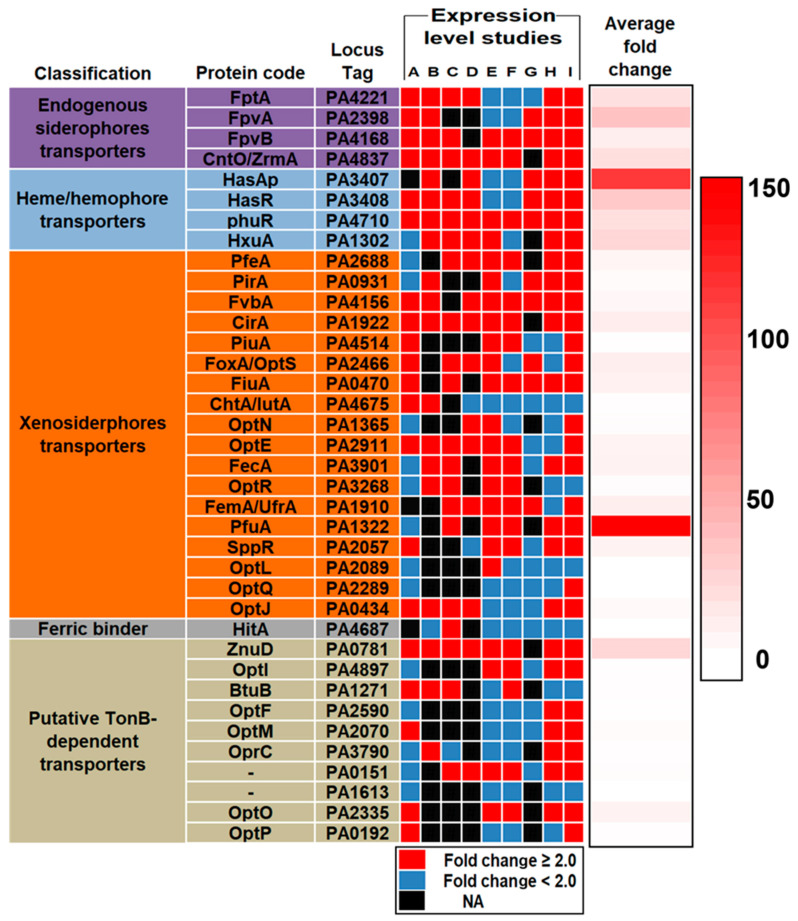
Heatmap showing the expression level studies and the average fold change for each IAP. White, light red, and dark red indicate low, moderate, and high expression levels, respectively.

*PfuA, hasAp, fpvA, hasR,* and *znuD* were the top five most highly expressed iron acquisition genes with average fold changes of 136, 105, 34, 31, and 23, respectively. *PfuA* was underexpressed compared to control in acute pneumonia infection in mice [25]. In human CF samples, *PfuA* showed an exceptionally high fold change (800) in one study [32] and a moderate fold change (3.5) in another study [28] compared to control. *PfuA* was overexpressed in chronic wound (5.4 folds) and acute burn (2.8 folds) infections compared to control [31], while it was underexpressed in chronic burn infections [28]. Damron et al. reported that *hasAp* was the most highly expressed gene in acute pneumonia infection in mice with a fold change of 339 compared to control [29]. In human CF samples, *hasAp* was 20 times more expressed than control [30]; however, it was underexpressed in the study of Cornforth et al. [28]. *HasAp* expression level was unparalleled by any other IAP in acute burn (133.8 folds), chronic wound (91.4 folds) infections in mice [31], and burn infections (256 folds) in humans [33]. However, it showed a low expression level also in the work of Cornforth et al. [28]. *FpvA* was expressed higher than other IAPs (71 folds) in acute pneumonia infection in mice [25]. It was 7 times more expressed than control in another study [29] with the same infection type and host. In CF human samples, *fpvA* was missing in two studies [30,32] and showed a low expression level in another study [28]. *FpvA* was 4 and 5 times more expressed than control in acute burn and chronic wound infections in mice, respectively [31]. *FpvA* was highly expressed (153 folds) in human burn infections, second to *hasAp*, in the study conducted by Gonzalez et al. [33]. *HasR* was not missing in any of the seven expression level studies reviewed in our analysis and showed moderate to high expression levels in all of the studies [25,29,30,31,32,33] except one [28]. In acute pneumonia infection in mice, it was 52 [25] and 79 [29] times more expressed than control. In CF human samples, it was 30 [32] and 8 [30] times more expressed than control. The same holds for acute burn (20 folds), chronic wound (39 folds) infections in mice [31], and human burn infections (47 folds) [33]. *znuD* was upregulated in all the reviewed expression level studies and was missing from only one study involving human burn infections [33]. In acute pneumonia infections in mice, *znuD* was 70 [25] and 32 [29] times more expressed than control. In CF human samples, fold changes were 30 [32], 5 [30], and 9 [28] compared to control. *znuD* showed a relatively small fold change (2) in acute burn infections [31] but a relatively high fold change (31) in chronic wound infections in mice in the same study. In addition, *znuD* was 6 times more expressed than control in human burn and wound infections in another study [28] (Table 2, Suppl. File S1, and Figure 1).

#### 3.1.2. Protein Antigenicity, Solubility and Conservation

PiuA, ChtA, FiuA, PfeA, and HitA were the top five proteins in terms of average antigenicity scores from Antigenic and AntigenPRO tools (Suppl. File S2). HitA, HasAp, ZnuD, PirA, and CntO were the most soluble according to solubility scores obtained from the SolArt tool (Suppl. File S2).

Twenty-eight out of thirty-seven IAPs scored higher than 90% mean IC score. OptL, SppR, OptN, OprC, and OptM were the top five most conserved proteins with mean IC scores between 99.6% and 99.8% and standard deviations below 0.5%. FvbA, FpvA, HitA, PiuA, and PA1271 came at the bottom of the list, where mean IC scores ranged from 50% to 86%, with FvbA showing the least conservation overall with a mean IC score of 50% ± 1.75% (Suppl. File S3).

No significant similarity to human or mouse proteomes was detected using default BLAST-P algorithm parameters (E-value threshold = 0.05). Increasing the E-value to 10 allowed slight similarities to appear where FpvB, HitA, HasAp, and PA0151 showed similarity to human proteome. PA1271, OptI, and PhuR showed similarity to mouse proteome, while FpvA and PA1613 showed similarity to both. However, a closer look at the alignments reveals that most of the similarities are short stretches of identical and/or functionally similar amino acids; about only five amino acids long.

#### 3.1.3. Venn Diagram Data Analysis

**Figure 2:**
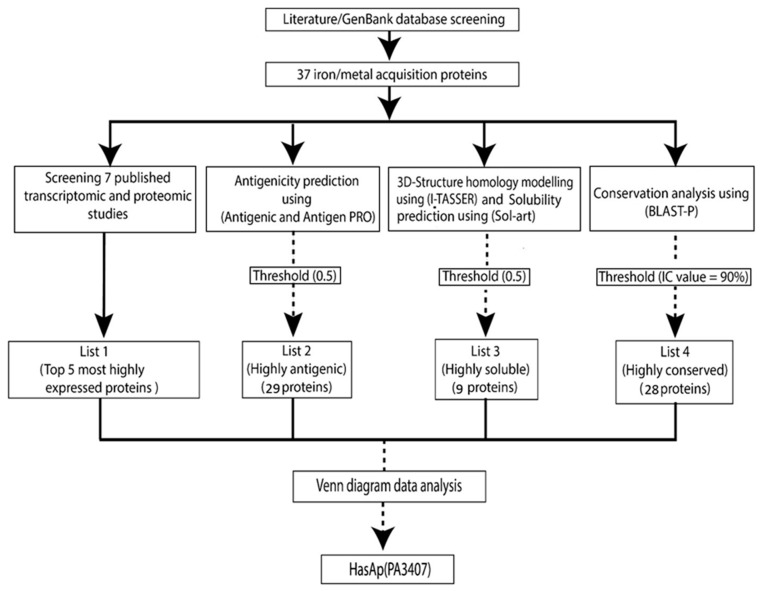
Flow chart describing the in silico selection of the candidate antigen.

**Figure 3:**
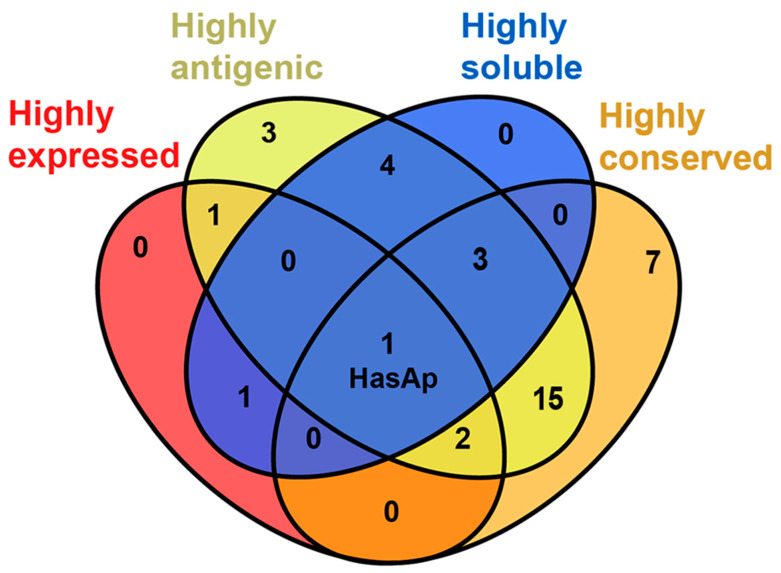
Venn diagram analysis showing that HasAp is shared between the four lists representing highly expressed, highly antigenic, highly soluble, and highly conserved IAPs.

List 1 included the top five most highly expressed proteins, PfuA, HasAp, FpvA, HasR, and ZnuD, according to average fold change (Suppl. File S1 and Figure 2). List 2 and 3 included 29 and 9 proteins whose average antigenicity and solubility exceeded the threshold (0.5), respectively (Suppl. File S2 and Figure 2). List 4 included 28 proteins which showed mean IC values exceeding the 90% threshold (Suppl. File S3 and Figure 2). Venn diagram data analysis showed that HasAp (PA3407) was shared between the four lists and therefore, it was considered the vaccine candidate of choice among IAPs (Figure 3 and Suppl. File S4).

### 3.2. SDS-PAGE and Western Blot Analysis

**Figure 4:**
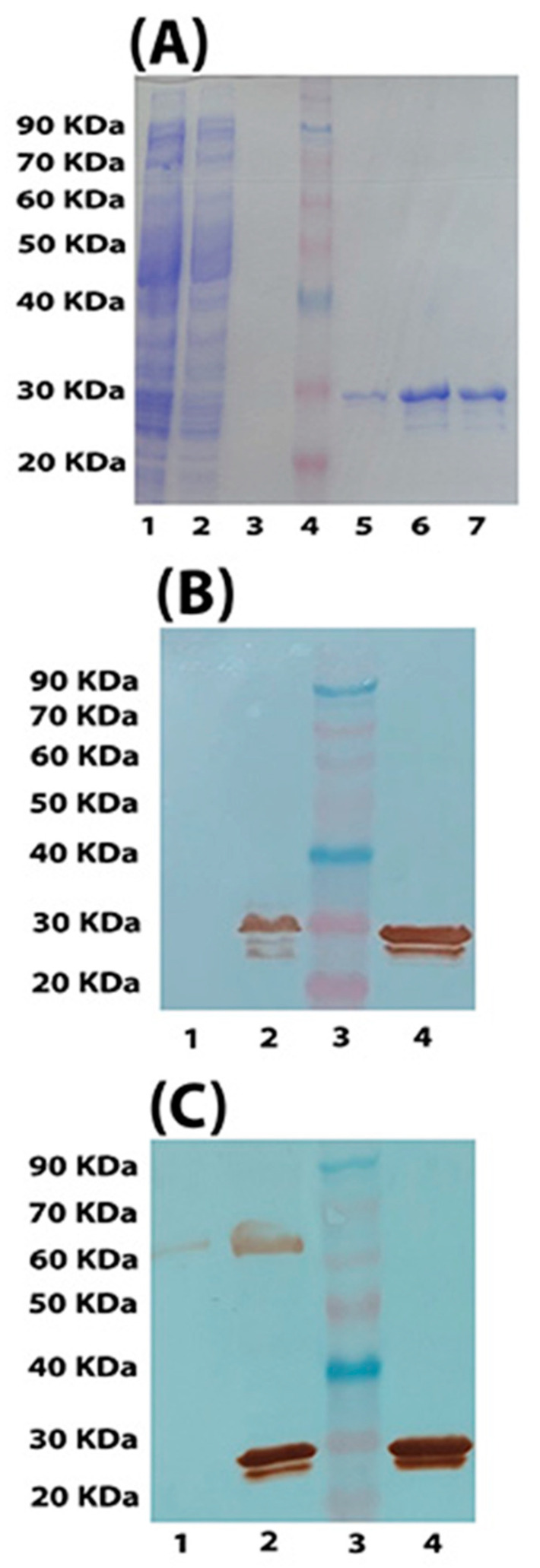
SDS-PAGE and Western blot of purified HasAp antigen. SDS-PAGE (**A**): Lane 1 is the induced culture lysate; lane 2 is the flow-through after passing the lysate through Ni-NTA agarose column; lane 3 is a sample from the second wash of the agarose resin; lane 4 is the pre-stained protein ladder; lanes 5, 6, and 7 are three successive eluates (E1 through E3) of purified His-tagged HasAp recombinant antigen (apparent molecular weight of 28 kDa). Western blot: The primary antibody is anti-His tag antibodies (**B**) or mouse polyclonal antibodies raised following sub-lethal dose injection of PA ATCC 9027 in mice (**C**). In both (**B**) and (**C**), lanes 1, 2, 3, and 4 represent non-induced lysate, induced lysate, pre-stained protein ladder, and HasAp protein band at an apparent molecular weight of 28 kDa, respectively. In (**C**), a non-specific, non-His-tagged protein band appears at an apparent molecular weight of 65 kDa.

A purified HasAp band was detected at an apparent size of 28 KDa (Figure 4A). Western blot analysis showed that purified HasAp reacted with primary anti-His tag antibodies (Figure 4B). In addition, it reacted with primary polyclonal antibodies collected from mice previously i.p.-immunized with sublethal doses of the same challenge strain (PA ATCC 9027) (Figure 4C), confirming that HasAp was utilized during infection and was immunogenic. A protein band was detected at an apparent molecular weight of 65 kDa, only when polyclonal primary antibodies were used (Figure 4C), indicating that this protein is non-His-tagged and non-specific.

### 3.3. HasAp Stimulates Antigen-Specific IgG in Immunized Mice

**Figure 5:**
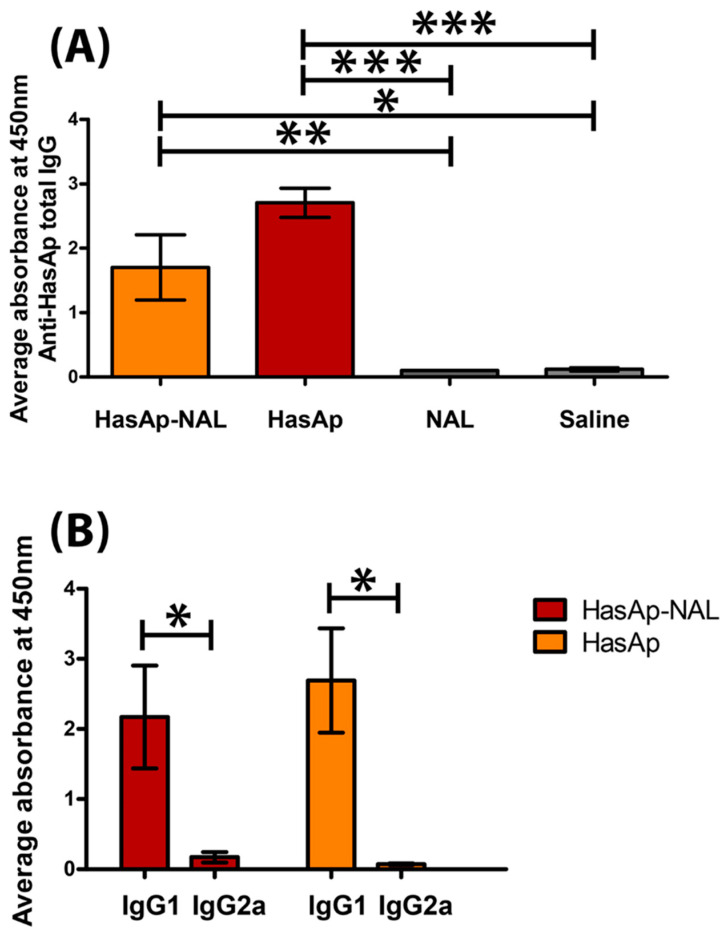
Indirect enzyme-linked immunosorbent assay (ELISA). Results represent absorbance readings at 450 nm averaged for all mice sera in each group. Wells were coated with purified HasAp antigen and primary antibodies were sera collected from mice 2 weeks after the fourth immunization before challenge, diluted 1/1000. Secondary antibodies were peroxidase-labeled anti-mouse IgG (total) (**A**) or anti-mouse IgG1 or IgG2a (**B**). Error bars represent mean ± SE (standard error of the mean). *: *p*-value ≤ 0.05, **: *p*-value ≤ 0.01, ***: *p*-value ≤ 0.001. One-way analysis of variance (ANOVA) was used, followed by Tukey’s test for post hoc analysis.

In antigen-specific ELISA (ASE), sera collected from HasAp-NAL- and HasAp-immunized mice two weeks after the last vaccine booster showed significantly higher reactivity with HasAp antigen compared to mice immunized with NAL (*p*-value ≤ 0.01 and *p*-value ≤ 0.001, respectively) or saline (*p*-value ≤ 0.05 and *p*-value ≤ 0.001, respectively) in total IgG antibody, respectively (Figure 5A). ASE also revealed that levels of antigen-specific IgG1 antibodies were significantly higher than IgG2a antibodies in HasAp-NAL- and HasAp-immunized mice (*p*-value ≤ 0.05) (Figure 5B). 

### 3.4. Quantification of Bacterial Load in Livers, Kidneys, and Lungs of Challenged Mice

**Figure 6:**
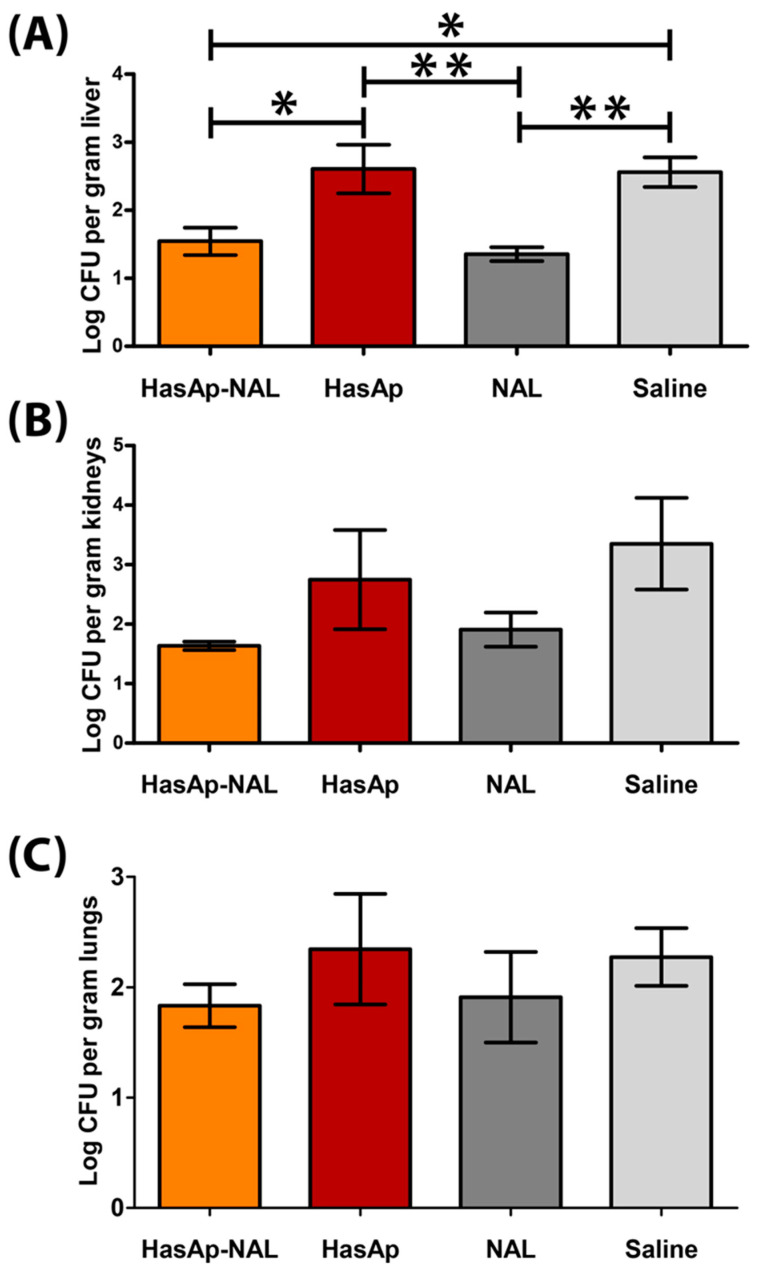
Bacterial burden of PA in livers (**A**), kidneys (**B**), and lungs (**C**) of challenged mice. Error bars represent mean ± SE (standard error of the mean). *: *p*-value ≤ 0.05, **: *p*-value ≤ 0.01. One-way analysis of variance (ANOVA) was used followed by Tukey’s test for post hoc analysis.

The bacterial load in the livers of HasAp-NAL group was significantly lower compared to HasAp and saline groups (*p*-value ≤ 0.05). Interestingly, no significant difference in bacterial count was observed between HasAp-NAL and NAL groups (*p*-value = 0.92) or between HasAp and saline groups (*p*-value = 0.99) (Figure 6A). 

The bacterial count in the kidneys of the HasAp-NAL group was also lower compared to HasAp and saline groups; however, the difference was not significant (*p*-value = 0.46 and 0.14, respectively). NAL group also showed a lower kidney bacterial burden than HasAp and saline groups, and the difference was not significant (*p*-value = 0.67 and 0.25, respectively). Likewise, no significant difference was observed either between HasAp-NAL and NAL groups (*p*-value = 0.97) or between HasAp and saline groups (*p*-value = 0.86) (Figure 6B). Similarly, in the lungs, the HasAp-NAL group showed a non-significant decrease in the bacterial burden compared to HasAp and saline groups (*p*-value = 0.74 and 0.81, respectively). Furthermore, similar to livers and kidneys, almost the same bacterial count was observed in the lungs of HasAp-NAL and NAL groups (*p*-value = 0.99) as well as HasAp and saline groups (*p*-value = 0.99) (Figure 6C).

### 3.5. Histopathology

**Figure 7:**
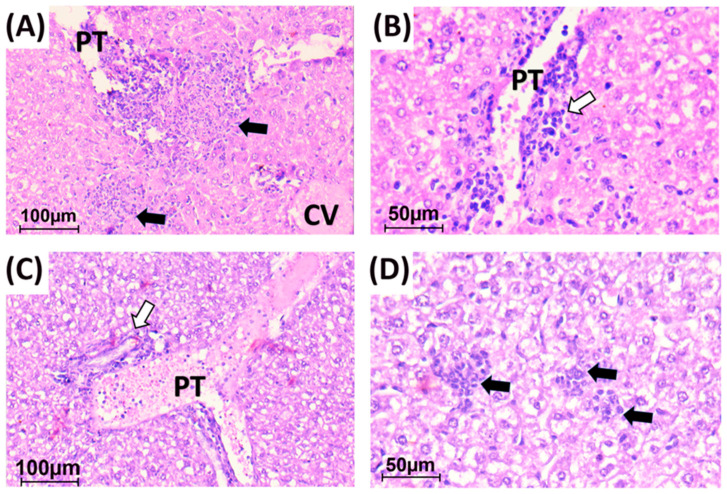

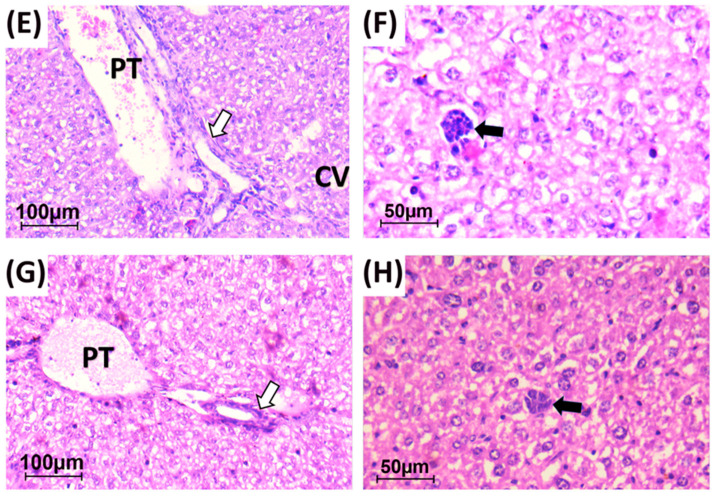
Histologic findings in H&E-stained liver sections in the studied groups: Saline: (**A**) Liver tissue showing mild distortion of lobular architecture. Portal tract (PT) exhibiting severe congestion of portal vessels and severe inflammation. Central veins (CV) are congested as well. Multiple large necroinflammatory foci (black arrows) were detected (×200). (**B**) High power view of one portal tract showing portal inflammation with interface hepatitis (white arrow) (×400). NAL: (**C**) Portal tract (PT) showing severe congestion of portal vessels and moderate inflammation (white arrow) (×200). (**D**) High power view showing multiple small necroinflammatory foci (black arrow) in liver lobule with diffuse degenerative changes of hepatocytes (×400). HasAp: (**E**) Portal tract (PT) showing severe congestion of portal vessels and still noted moderate inflammation (white arrow) (×200). (**F**) High power view showing small necroinflammatory foci (black arrow) among degenerated hepatocytes (×400). HasAp+NAL: (**G**) Portal tract (PT) showing minimal inflammation and moderate congestion of portal vessels (white arrow) (×200). (**H**) High power view showing small infrequent necroinflammatory foci (black arrow) and only focal degenerative changes in hepatocytes (×400). PT: portal tract. CV: central vein. Black arrow: necrotic focus. White arrow: portal inflammation.

PA-infected mice which received saline showed a distortion of liver architecture with diffuse degenerative changes in hepatocytes. Hepatocytes were ballooned with feathery granular cytoplasm. Portal tracts showed moderate to severe inflammation. Inflammatory cells were composed mainly of neutrophils admixed with lymphocytes and plasma cells. Interface hepatitis was frequently seen. Hepatic lobules showed numerous necroinflammatory foci. They were large-sized with the formation of microabscesses especially in subcapsular zones. Central veins and portal vessels showed severe congestion. They were dilated and ectatic with intraluminal RBCs (Table 3 and Figure 7A,B). 

The group which was pretreated by NAL showed slightly better hepatic histology compared to the saline group. Although hepatocytic degenerative changes were still detected, portal inflammation was only mild to moderate. Interface hepatitis was focally seen. Necroinflammatory foci were still detected but were smaller and less in number compared to the saline group. Vascular congestion was moderate (Table 3 and Figure 7C,D). 

Similar improvement was seen in HasAp pretreated mice. The portal inflammation was mild to moderate and seen only in some portal tracts. Necroinflammatory foci tended to decrease in number and to be smaller in size when compared to saline-treated group. Vascular congestion was significantly milder than in the saline-treated group. Hepatocytic degenerative changes were still seen (Table 3 and Figure 7E,F). 

Meanwhile, mice pretreated by HasAp-NAL combination showed minimal portal inflammation. Necroinflammatory foci were infrequent and hardly seen where the histopathology score (score 1) was significantly lesser than the NAL group and saline-treated group (*p*-value ≤ 0.05). However, mild to moderate vascular congestion was still seen in most areas (Table 3 and Figure 7G,H).

HasAp-NAL-immunized mice showed a significantly milder hepatocyte necrosis compared to NAL and saline groups (*p*-value ≤ 0.05). Vascular congestion was significantly lesser in HasAp-immunized mice compared to the saline group (*p*-value ≤ 0.01). Scoring was performed as previously described [24,51].

## 4. Discussion

*P. aeruginosa* is a ferocious superbug predominantly causing nosocomial infections usually associated with compromised immunity in patients with neutropenia, CF, or severe burns [1]. Because PA is a multi-drug resistant pathogen [53] with remarkable adaptability and versatility in antibiotic resistance mechanisms [2], it is likely that all available antibiotics will be ineffective and vaccination will be an important approach to control infections caused by this pathogen [5]. To date, there is no clinically available licensed vaccine against *P. aeruginosa* despite the efforts of the scientific community over the past decades [54].

Iron is a first-row transition metal that is indispensable for the existence of both prokaryotes and eukaryotes [7]. PA relies heavily on this metal for growth and colonization to such an extent that 6% of this pathogen’s transcriptome is iron-associated [55]. However, the host’s iron is not freely available but rather mostly bound to heme as well as proteins such as transferrin and lactoferrin [56]. Therefore, this pathogen engages in a vicious battle for the host’s iron by employing multiple mechanisms of iron acquisition, including siderophore piracy, heme utilization, and direct iron uptake [15]. 

Previous studies have evaluated the protective efficacy of some IAPs. FpvA, HasR, and PhuR whole proteins, alone or in combination, were not protective against acute murine pneumonia when combined with curdlan immunoadjuvant [25]. However, FpvA peptides conjugated to keyhole limpet hemocyanin (KLH) carrier and combined with curdlan protected mice from acute pneumonia caused by PA [26]. In addition, HitA combined with Bacillus Calmette–Guerin (BCG) and incomplete Freund’s adjuvant (IFA) immunoadjuvants protected mice from acute PA infection [24]. Because of the undisputed importance of iron in the growth and colonization in the host as well as the promising protective effect of some IAPs when tested as vaccine targets [24,25,26,27], we have been motivated to perform a comprehensive in silico analysis of IAPs of PA, and to meticulously select a candidate vaccine target for in vivo evaluation. We proposed that the candidate antigen should possess multiple characteristics. First, it should be highly expressed in different types of infections. High expression of a certain protein during infection indicates that this protein is essential for virulence because this means PA allocates expensive metabolic resources to maintain its expression. Second, it should be highly antigenic. Third, because the formation of insoluble inclusion bodies significantly decreases protein yields and disrupts protein folding [57], high solubility of the candidate antigen is desirable to make the recombinant protein purification process as straightforward as possible. This is important from a pharmaceutical manufacturing perspective, where upon overproduction of highly soluble proteins, high yields are achieved in a native conformation, leading to a more reliable immune response. Therefore, there is no need for time- and money-consuming re-solubilization/refolding processes that would complicate large-scale production of the vaccine. Fourth, the candidate antigen should be well-conserved across PA strains. Finally, it should not possess significant similarity to human or mouse proteins because it would be poorly immunogenic (self-antigen) in these hosts or even might induce auto-immunity [23,58].

In this study, we examined seven previously published transcriptomic and proteomic analyses to assess the level of expression of IAPs in different types of infection: acute pneumonia in mice [25,29], human CF infections [28,30,32], and burn/wound infections in mice or human [28,31,33]. We used the average fold change in expression level compared to control as a metric to rank IAPs. Although *pfuA* had the highest average fold change (136 ± 325), a very high level of expression (800 folds) was noticed in only one study [32], which sharply skewed the mean. *HasAp* came second after *pfuA*, being expressed 105 times more than the control, where high expression was more consistent across several studies [29,30,31,33] with lower standard deviation (±130). Moreover, *hasAp* was expressed higher than any other protein, whether IAP or not, in acute pneumonia infection in mice [29]. It was also expressed higher than any other IAP in acute burn (133.8 folds), chronic wound (91.4 folds) infections in mice [31], and burn infections (256 folds) in humans [33]. Among the top 10 most highly expressed genes, *fpvA, hasR*, and *phuR* came in the third, fourth, and eighth positions, respectively, in line with previous studies which stressed the importance of these proteins in virulence and colonization [19,26,27,59]. Our in silico analysis also highlighted the high level of expression of other IAPs which have not been tested as vaccine targets, yet came in the top 10 list. These proteins are ZnuD, HxuA, CntO, FptA, and CirA, which came in the fifth, sixth, seventh, ninth, and tenth positions and achieved 23.2-, 23-, 18.7-, 18.1-, and 12-fold changes, respectively. CntO, or ZrmA, as named in some studies [60,61], is a receptor for a metallophore called pseudopaline that was proven to be involved in the transport of mainly zinc as well as several other divalent metals including iron, cobalt, and nickel [60,61,62]. Interestingly, ZrmA PA mutant had significantly decreased extracellular protease activity as well as reduced virulence [60]. ZnuD, named after a similar protein that is responsible for zinc acquisition in *Neisseria meningitidis* [63], together with ZrmA, were found to be significantly upregulated as a response to zinc depletion in PA, indicating a probable role in zinc acquisition [64]. Whether ZnuD participates in iron acquisition in PA is yet to be ascertained. 

Two IAPs, FpvA and HitA, previously tested as vaccine targets [24,26], achieved very low conservation scores in our analysis. The fact that FpvA is a poorly conserved protein is backed by previous research which shows that the *fpvA* gene locus is the most variable locus in the PA genome [65,66]. Such a fact was overlooked during the bioinformatic selection of FpvA peptides in the work of Sen-Kilic et al., where the selection was based only on high immunogenicity and solubility and low homology to proteins of human and commensal bacteria [26]. Consequently, a tempting strategy would be to use only conserved epitopes from these proteins, ideally combined with epitopes from other IAPs [67].

Venn diagram analysis revealed that HasAp was the only IAP that met all our selection criteria, being very highly expressed, highly antigenic, soluble, and conserved. HasAp is a protein secreted by PA which binds extracellular iron-loaded heme and traffics it back into PA through the HasR surface receptor [19,59]. Analogues of the hemophore HasAp also exist in other Gram-negative bacteria such as *Serratia marcescens* and *Yersinia pestis* [68].

A recent review reported that either a mixed Th1/Th2 or Th1/Th17 immune response is critical for protection against PA infections, while a Th2-skewed immune response is associated with inefficient protection [69]. Therefore, to validate the protective efficacy of HasAp as a sub-unit vaccine, we carried out an animal trial using a novel immuno-adjuvant, NAL [34]. NAL has been successfully used, combined with the DNA vaccine against herpes simplex virus type 1, to stimulate a robust Th1-dominated cellular immunity characterized by a high IgG2a/IgG1 ratio [34]. Another study used NAL in combination with alum, another adjuvant that stimulates the Th2 arm of the cellular immunity, to induce a balanced Th1/Th2 immune response. In the latter study, the protective efficacy of recombinant pilin protein (rPilA), the building block of type IV pili responsible for twitching motility in PA, was investigated. Mice immunized with r-PilA-NAL-alum had higher levels of IFN-gamma, IL-17, and IgG2a, sera with higher opsonic activity and lower bacterial loads compared to mice immunized with r-PilA-NAL or any of the controls (r-PilA, NAL, or alum) [70]. However, we hypothesized that alum is dispensable because of several reasons. First, NAL alone could enhance the r-PilA protective efficacy. For example, mice in the r-PilA-NAL group showed 66.6% and 75% survival rates when challenged with PA PAO1 strain or PA clinical isolate, respectively, which is comparable to the r-PilA-NAL-alum group (75% in both strains). Survival rates of mice in the r-PilA-NAL group were significantly higher than any of the control groups when challenged with PA clinical isolate and higher than PBS and NAL groups when challenged with PA PAO1 strain. Moreover, the lymphocyte stimulation index as well as IFN-gamma, IL-17, and IgG2a levels were significantly higher in the r-PilA-NAL group compared to control groups [70]. Second, aluminum adjuvants are not as safe as generally believed according to several papers which reported multiple toxic effects caused by aluminum adjuvants such as autoimmune disorders, brain inflammation, autism, and other neurological complications [71,72]. In fact, combining alum with a Th1-inducer (NAL) further amplifies the Th1 immune response, increasing the risk of autoimmune disease [71,73]. On the other hand, NAL appears to be generally safe, and to our knowledge, no serious toxic effects have been reported, especially at low doses [74]. Third, alum preparation is a lengthy procedure requiring multiple steps and reagents [75]. Therefore, we hypothesized that NAL adjuvant only combined with HasAp antigen would yield a mixed Th1/Th2 immune response, the type of immunity required for protection against PA infections [69].

ELISA results showed that IgG from sera of HasAp-NAL- and HasAp-immunized mice had a significantly higher reactivity with HasAp antigen compared to mice immunized with NAL or saline groups (Figure 5A). However, a large fraction of this reactivity was due to IgG1 rather than IgG2a antibodies (Figure 5B) even in the HasAp-NAL group, contrary to what was expected from NAL being a Th1-inducer [34,48,70,76]. 

Intriguingly, the bacterial load in livers of HasAp-NAL- and NAL-immunized mice was significantly lower than in the HasAp or saline groups. A similar trend was observed in lungs and kidneys, where the bacterial load was lower in both HasAp-NAL and NAL groups, although the difference was not statistically significant. 

Surprisingly, no significant difference in bacterial counts was observed in livers, lungs, or kidneys of either HasAp-immunized mice when compared to the saline group or of HasAp-NAL when compared to the NAL group. However, the histopathological examination revealed significantly lesser hepatocyte necrosis in the HasAp-NAL group and also less vascular congestion in HasAp-immunized mice compared to the saline group (Table 3). 

Several hypotheses might explain why HasAp did not provide the anticipated protection. First, knowing that the Th2-dependent immune response is directed towards external threats [77], it could be that HasAp, being extracellular [19,59,78], is intrinsically a Th2-inducing antigen, especially as Firacative et al. have recently identified Th1- and Th2-specific antigens in *Cryptococcus neoformans* [79]. It is well established in the literature that a Th2-oriented immune response is not optimal for protection against PA infection [69]. Indeed, a recent study showed that HitA, an IAP which was found in the biofilm secretome of PA [80], combined with BCG and IFA adjuvants, produced a mixed Th1/Th2 immune response and protection against PA acute infection [24]. A possible explanation for this apparent discrepancy is that the immunomodulatory effects of BCG (Th1-directing) and IFA (Th2-directing) resulted in a favorable mixed Th1/Th2 outcome despite the extracellular nature of HitA. Second, our analysis shows that PA uses multiple tools for iron acquisition (Figure 2 and Suppl. File S1) [20], including two other heme acquisition systems, PhuR and HxuA, beside the HasAp-HasR system [19,59]. Therefore, blocking HasAp alone might not significantly affect the iron acquisition capacity of PA. Third, we used a different route of administration and model of infection compared to those applied by the studies used to assess the level of HasAp expression. HasAp was shown to be highly expressed in the context of acute murine pneumonia achieved through intranasal inoculation of PA [29], acute burn/chronic wound infections in mice [31], and burn infections in humans [33]. However, in our study, we performed an acute infection in mice through i.p. injection of PA. Therefore, the high expression levels of HasAp in the mentioned studies may not be extrapolatable to our study if HasAp expression was sensitive to the route of administration (intranasal versus i.p.), the type of infection (acute pneumonia/chronic pneumonia/burn infection/wound infection), or the host (mouse/human).

Because of the low IgG2a/IgG1 ratio in the HasAp-NAL-treated group as well as the absence of a significant difference in bacterial count between the HasAp-NAL- and NAL-treated groups, we proposed that protection in NAL-treated groups was not due to an adaptive immune response but rather through an effect mediated by innate immunity. Indeed, it was reported that opioid receptor antagonists, e.g., NAL, at low doses, enhance the release of endogenous opioids, as well as upregulate opioid receptors [81,82]. Interestingly, low-dose NAL administered to a patient under sufentanil-controlled analgesia increased levels of opioid growth factor (OGF), which is an endogenous opioid, and natural killer (NK) cells, a critical player in innate immunity [83,84]. Other studies also showed that OGF increases numbers of NK cells [85,86,87]. The critical role that NK cells play in innate immune response is well established in the literature. NK cells are responsible for the early release of an array of cytokines which coordinate the innate immune response such as IFN-gamma, GM-CSF (granulocyte macrophage colony-stimulating factor), IL-5, IL-6, IL-10, transforming growth factor (TGF)-β, IL-12, IL-13, IL-16, IL-17, and IL-22 [83]. Mice immunized with an *aroA* deletion mutant of PA strain PA14 were protected, in an antibody-independent manner, against acute murine pneumonia caused by LPS-heterologous PA strains. Intriguingly, IL-17 production was pivotal in achieving such protection [88]. Moreover, human NK cells kill PA through direct contact via the release of granzymes B and H [89]. In addition, NK cells, mainly through an effect mediated by the NKG2D ligand, have been shown to help in the resolution of PA acute lung infection [90]. NK cells release antimicrobial peptides such as α-defensins and cathelicidin (or LL37) in humans [91]. Human NK cells, through Toll-like receptors (TLRs) expressed on their surface and the antimicrobial peptides released by these cells, such as α-defensins, have been shown to directly interact with and kill *Klebsiella pneumoniae* [92]. Importantly, NK cells release a host of chemokines which direct immune cells to the site of infection [83,93].

It is important to note that the current study has some limitations. The protective efficacy of HasAp could have been enhanced by testing more immunization regimens, more doses of both HasAp and NAL, different immunization/challenge routes, as well as different adjuvants such as curdlan [26], IFA [94], or IFA/BCG combination [24]. In addition, despite the reported toxicity of alum [71,72], using lower doses of alum combined with NAL might have achieved a balanced Th1/Th2 immune response [70] with reduced or diminished alum toxicity.

Finally, future research trials should focus on evaluating combinations of HasAp together with other whole-protein IAPs such as PhuR and HxuA to block the heme acquisition pathway. Furthermore, using peptide vaccines [95] composed of peptides selected from the top ten most highly expressed IAPs, provided in our analysis, is another possible approach.

To conclude, we have provided a detailed in silico analysis of IAPs of PA followed by in vivo evaluation of the best IAP, HasAp. Although HasAp did not provide the anticipated efficacy, it should be further evaluated as a vaccine candidate using different immunization regimens and different models of infection, preferably combined with other IAPs. Our study revealed that the NAL immuno-adjuvant caused a noticeable stand-alone protection which could be attributed to the stimulation of innate immunity. Further assessment of the usage of NAL as an immune booster, alone or in combination with antibiotics, seems to be appealing. The current study also sheds light on several highly expressed IAPs as well as zinc and other divalent metal transporters whose efficacy as vaccine targets should be subjected to further evaluation. 

## Figures and Tables

**Table 1:** Summary of the used animal immunization protocol.

Animal Group	Immunization (S.C.) on Days 0, 14, 28, and 77
HasAp-NAL	HasAp (10 μg/mouse) plus Naloxone (150 μg/mouse)
HasAp	HasAp (10 μg/mouse)
NAL	Naloxone (150 μg/mouse)
Saline	Saline

**Table 2:** Proteomic studies used to assess the expression levels of IAPs of PA.

	Authors	Technique Used for Quantification of Expression Level	Infection Type and Host	Fold Change Relative to	Reference
**A**	Liu et al.	Quantitative real-time PCR	Acute pneumonia in mice	*rpsL* internal control	[25]
**B**	Damron et al.	RNA-seq analysis	Acute pneumonia in mice	In vitro (PIA)	[29]
**C**	Wu et al.	Proteome analysis (mass spectrometry)	Human CF sputum samples	Ex vivo (LB)	[32]
**D**	Rossi et al.	RNA-seq analysis	Human CF sputum samples	Ex vivo (LB)	[30]
**E**	Cornforth et al.	RNA-seq analysis	Human CF sputum samples	In vitro (CDM)	[28]
**F**	Cornforth et al.	RNA-seq analysis	Human wound and burn infections	In vitro (CDM)	
**G**	Gonzalez et al.	RNA-seq analysis	Human burn infections	BWE vs. LB pH 9.0	[33]
**H**	Turner et al.	RNA-seq analysis	Acute burn infections in mice	In vitro (MOPS-succinate)	[31]
**I**	Turner et al.	RNA-seq analysis	Chronic wound infections in mice	In vitro (MOPS-succinate)	

PIA: *Pseudomonas* isolation agar; LB: Luria Bertani medium; CDM: chemically defined media; BWE: human burn wound exudate; MOPS-succinate: a minimal growth medium buffered by MOPS (morpholine–propanesulfonic acid) and supplemented with succinate as the only carbon source.

**Table 3:** Median values of histopathology scores of livers isolated from immunized and non-immunized mice.

Group	Hepatocyte Necrosis (Necroinflammatory Foci and Microabcesses) (0–4)	Portal Tract Inflammation (0–3)	Vascular Congestion (0–3)
HasAp-NAL	1 *	1	2
HasAp	1	1.5	1 **
NAL	2	2	2
Saline	2.5	2.5	3

*: *p*-value ≤ 0.05, **: *p*-value ≤ 0.01 by Kruskal–Wallis test and Dunn’s multiple comparison test.

## Data Availability

Publicly available datasets were analyzed and included in this study.

## Supplementary Materials

The following supporting information can be downloaded at: https://www.mdpi.com/article/10.3390/vaccines11010028/s1, Suppl. File S1 (spreadsheet): Expression level studies. Suppl. File S2 (spreadsheet): Antigenicity and solubility data. Suppl. File S3 (spreadsheet): Conservation analysis of antigens in PA strains. Suppl. File S4 (spreadsheet): Venn diagram data. Suppl. File S5: Uncropped SDS-PAGE and western blots.
