# Supplementary material for: Iron Acquisition Proteins of Pseudomonas aeruginosa as Potential Vaccine Targets: In Silico Analysis and In Vivo Evaluation of Protective Efficacy of the Hemophore HasAp
Source: Vaccines (Basel). 2022 Dec 23;11(1):28. doi: 10.3390/vaccines11010028 (PMC9864456; doi:10.3390/vaccines11010028)
Supplement: Supplementary file 1 [file vaccines-11-00028-s001.zip › Suppl file S1- Expression level studies.pdf]

Classification	Protein code
Endogenous siderophores transporters	FptA
	FpvA
	FpvB
	CntO/ZrmA
Heme/hemophore transporters	HasAp
	HasR
	phuR
	HxuA
Xenosiderphores transporters	PfeA
	PirA
	FvbA
	CirA
	PiuA
	FoxA/OptS
	FiuA
	ChtA/IutA
	OptN
	OptE

	FecA
	OptR
	FemA/UfrA
	PfuA
	SppR
	OptL
	OptQ
	OptJ
Ferric binder	HitA
Putative TonB-dependent transporters	ZnuD
	OptI
	BtuB
	OptF
	OptM
	OprC
	-
	-
	OptP
	OptO

**All expression level data represent non-
**Data from Liu et al. study represent aver
**Log2 fold changes from Rossi et al. stuc

Expression level

Locus Tag	A	B	C	D	E	F
PA4221	3.3	41.9	3.7	53.1	1.58	0.89
PA2398	71	7.4			0.08	0.05
PA4168	2.2	5.2	18		16.7	1.56
PA4837	8.6	15	16	21.1	17.1	2.64
PA3407		339		19.7	0.29	0.41
PA3408	52	79	30.4	8	1.96	1.77
PA4710	22	29	7.3	27.9	17.1	1.73
PA1302	1.5	6.3	136	14.9	17.1	0.87
PA2688	1.9		22	2.6	8.57	4.59
PA0931	0.9	3.1			3.25	1.23
PA4156	2.6	4.8		3.1	3.25	3.03
PA1922	21	8	11	8.57	14.9	5.66
PA4514	5.2				3.03	1.41
PA2466	2.2		39	4.44	4.59	0.99
PA0470	2.3		8.4		4.03	3.48
PA4675	6.6	6.9		0.13	0.76	0.93
PA1365	1.7			5.66	4.29	1.51
PA2911	12	3.6	6.2	8.57	18.4	13.9

**PA3901**	1.2	40	4.1		3.73	1.41
**PA3268**	1.5	6.9	7.1		3.01	2.14
**PA1910**			48	13	13	1.57
**PA1322**	1.9		800		3.48	1.59
**PA2057**	2.4			0.23	2.71	3.1
**PA2089**	1.2				2.14	1.25
**PA2289**	0.9				0.89	1.34
**PA0434**	12	5.6	2.7	6.06	1.24	1.15
**PA4687**		1.99	3.5		0.93	0.98
**PA0781**	70	32	30	5.28	9.13	5.66
**PA4897**	1.3				5.28	1.43
**PA1271**	4.5	6.6	2.1		1.27	2.11
**PA2590**	1.3				1.1	0.44
**PA2070**	2.7				1.73	1.48
**PA3790**	1.2	3.3	0.59		0.47	0.55
**PA0151**	1.9		2.8	2.46	3.25	1.73
**PA1613**	1.7				1.46	0.87
**PA0192**	3.2				1.47	0.84
**PA2335**	9				5.66	11.3

Fold change ≥ 2.0

Fold change < 2.0

NA

log scaled fold changes.
rage of fold changes of all intervals from both strai
dy were inverted to obtain non-log scaled data. The

studies

G	H	I	Average fold change per IAP	± Standard deviation
-5.6	38.6	25.6	18.125346	21.85
153	3.65	5.37	34.358203	58.11
36.9	3.33	5.79	11.213453	12.17
	4.2	65	18.71079	19.80
256	134	91.4	105.12424	133.08
47.3	20	39	31.013951	26.01
52.2	6.28	4.8	18.708701	16.17
	3.6	3.5	22.983299	46.07
	3.4	5.3	6.904733	7.01
10.3	3.3	3	3.5710329	3.12
3.39	5.8	18.2	5.5171589	5.24
	4.8	23.5	12.206776	7.03
-2.1	1.04	3.3	1.9704189	2.50
28.8	1.9	10.8	11.588555	14.34
5.6	3.9	34.3	8.8611062	11.39
1.49	0.6	0.7	2.2621907	2.79
	1.6	3	2.9572121	1.71
-3.1	0.8	3.1	7.0972438	6.85

-1.3	4.2	11.9	8.1491879	13.44
	0.44	0.6	3.1050288	2.80
5.56	1.7	4.5	10.914633	16.38
	2.8	5.4	135.8577	325.36
-1.2	14.9	34.1	8.0413733	12.62
1.32	1.5	1.88	1.544531	0.39
-1.3	2	5	1.4601381	2.07
-1.1	2.5	4.6	3.8334406	3.73
1.18	0.57	0.37	1.1905263	1.08
	2.1	31.1	23.184093	22.89
-2.4	2.7	4.3	2.1053227	2.69
	0.22	0.2	2.4335984	2.35
1.53	3.2	5.5	2.1790193	1.87
1.83	4.3	8.15	3.3600268	2.56
	4.67	6.95	2.5347584	2.53
1.32	3.6	6.12	2.9030636	1.51
	0.8	0.8	1.1225353	0.42
	2	5.36	2.5693585	1.78
	2.89	13.4	8.4618625	4.25

ns

e average of exponential and stationary phases w

vas used, except for PA4675 and PA0434, wher

e the data represent fold changes in either exponen

ntial or stationary phases, respectively.
