# Supplementary material for: Iron Acquisition Proteins of Pseudomonas aeruginosa as Potential Vaccine Targets: In Silico Analysis and In Vivo Evaluation of Protective Efficacy of the Hemophore HasAp
Source: Vaccines (Basel). 2022 Dec 23;11(1):28. doi: 10.3390/vaccines11010028 (PMC9864456; doi:10.3390/vaccines11010028)
Supplement: Supplementary file 1 [file vaccines-11-00028-s001.zip › Suppl file S2- Antigenicity and solubility data.pdf]

Color

Indication
Pyoverdine receptor
heme utilization protein
Xenosiderophore transporter
Ferric binder
Putative TonB-dependent transporter
Higher than threshold (0.5)
Lower than threshold (0.5)
