# Supplementary material for: Iron Acquisition Proteins of Pseudomonas aeruginosa as Potential Vaccine Targets: In Silico Analysis and In Vivo Evaluation of Protective Efficacy of the Hemophore HasAp
Source: Vaccines (Basel). 2022 Dec 23;11(1):28. doi: 10.3390/vaccines11010028 (PMC9864456; doi:10.3390/vaccines11010028)
Supplement: Supplementary file 1 [file vaccines-11-00028-s001.zip › Suppl. File S4- Venn diagram data.pdf]

[illegible]		
**Highly expressed, antigenic, conserved and soluble**	**Highly expressed + Highly antigenic**	**Highly expressed + Highly soluble**
HasAp	FpvA	ZnuD

Venn diagram data summary

Highly expressed, antigenic and conserved	Highly antigenic	Highly antigenic and highly soluble	Highly antigenic, soluble and conserved
PfuA	FptA	PiuA	FoxA
HasR	OptR	HitA	OptN
	BtuB	FvbA	CntO/ZrmA
		PirA	

Highly antigenic and highly conserved	Highly conserved
ChtA	FemA
FiuA	CirA
PfeA	PA1613
PhuR	OptF
OptQ	SppR
FpvB	OptL
OptE	OptO
FecA	
