# Supplementary material for: Iron Acquisition Proteins of Pseudomonas aeruginosa as Potential Vaccine Targets: In Silico Analysis and In Vivo Evaluation of Protective Efficacy of the Hemophore HasAp
Source: Vaccines (Basel). 2022 Dec 23;11(1):28. doi: 10.3390/vaccines11010028 (PMC9864456; doi:10.3390/vaccines11010028)
Supplement: Supplementary file 1 [file vaccines-11-00028-s001.zip › Suppl. File S5- Uncropped SDS-PAGE and western blots.pdf]

(A)

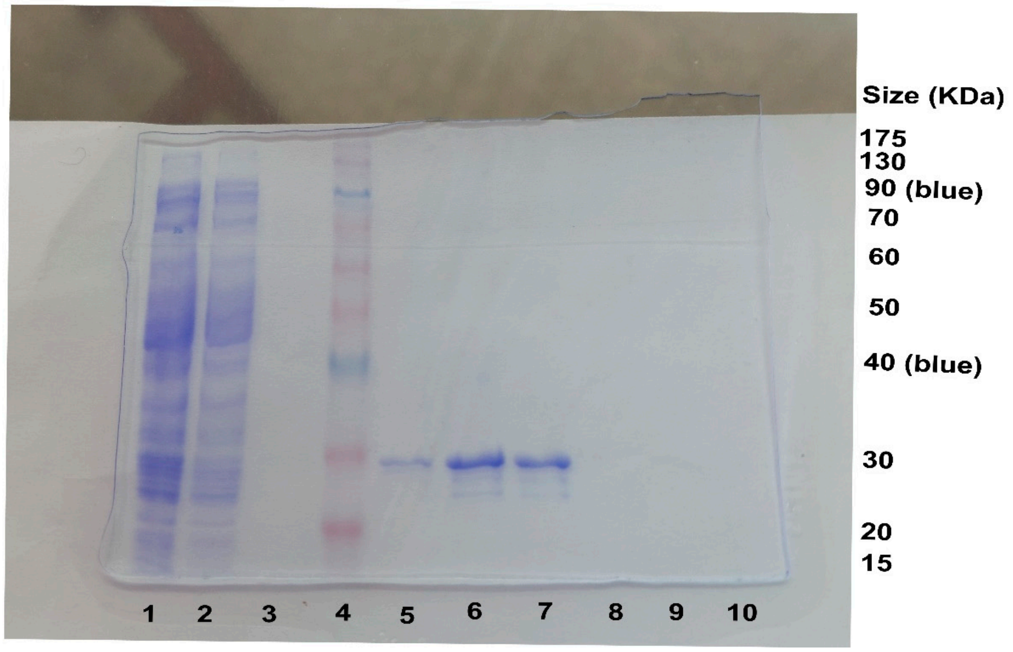

SDS-PAGE and Western blot of purified HasAp antigen. SDS-PAGE (**A**): Lane 1 is the induced culture lysate; lane 2 is the flow-through after passing the lysate through Ni-NTA agarose column; lane 3 is a sample from the second wash of the agarose resin; lane 4 is the pre-stained protein ladder; lanes 5, 6, and 7 are three successive eluates (E1 through E3) of purified His-tagged HasAp recombinant antigen (apparent molecular weight of 28 kDa); lanes 8, 9, 10 are empty.

(B)

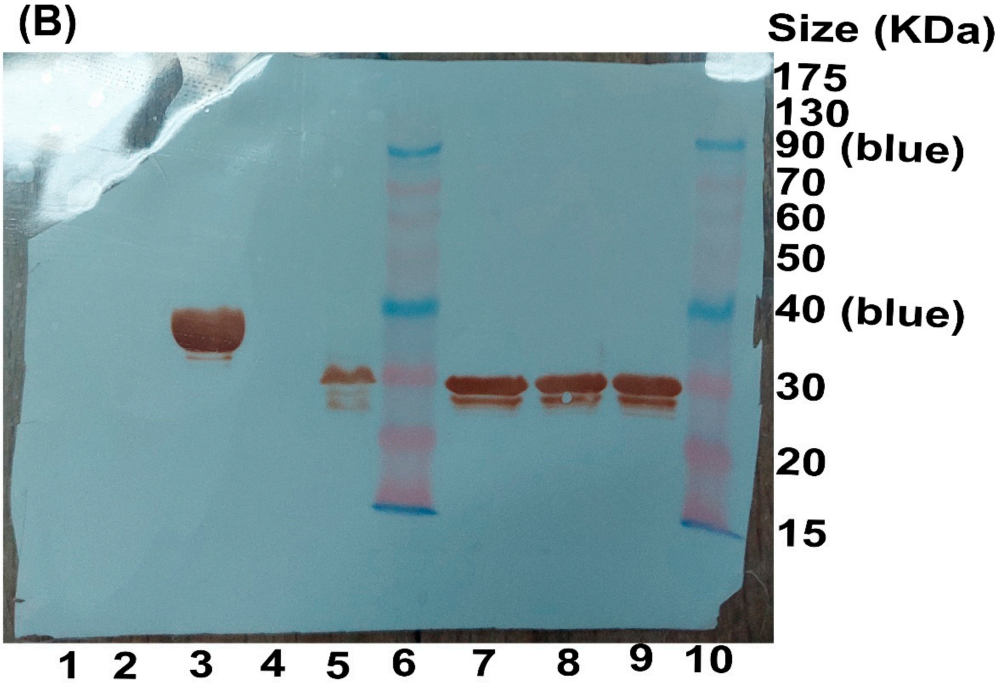

(C)

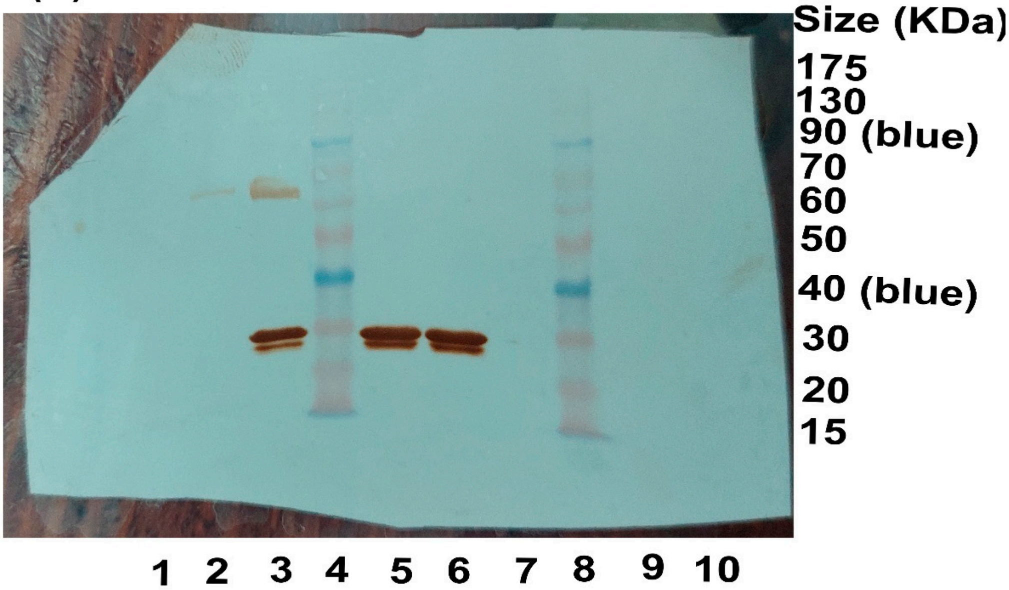

Western blot: The primary antibody is anti-His tag antibodies (**B**) or mouse polyclonal antibodies raised following sub-lethal dose injection of PA ATCC 9027 in mice (**C**). In (**B**), lanes 1 and 2 are empty, lanes 3 represents an irrelevant his-tagged protein, lanes 4,5,6,7,8,9,10 represent non-induced lysate, induced lysate, pre-stained protein ladder, three HasAp protein bands at an apparent molecular weight of 28 kDa and another protein ladder, respectively. In (**C**), lanes 2, 3 represent non-induced and induced lysates,

respectively. Lanes 4, 8 represent pre-stained protein ladder. Lanes 5, 6 represent HasAp elutions while lanes 1, 7, 9 and 10 are empty. In (**C**), lanes 2 and 3 show a non-specific, non-His-tagged protein band at an apparent molecular weight of 65 kDa,
